# eXamine: Visualizing annotated networks in Cytoscape

**DOI:** 10.12688/f1000research.14612.2

**Published:** 2018-07-05

**Authors:** Philipp Spohr, Kasper Dinkla, Gunnar W. Klau, Mohammed El-Kebir

**Affiliations:** 1 Algorithmic Bioinformatics, Heinrich Heine University, Düsseldorf, Germany; 2 Independent Researcher, Eindhoven, Netherlands; 3 Department of Computer Science, University of Illinois at Urbana-Champaign, Urbana, IL, USA

**Keywords:** functional subnetwork modules, visualization, enrichment, graph drawing, network community, Euler diagram

## Abstract

eXamine is a Cytoscape app that displays set membership as contours on top of a node-link layout of a small graph. In addition to facilitating interpretation of enriched gene sets of small biological networks, eXamine can be used in other domains such as the visualization of communities in small social networks.

eXamine was made available on the Cytoscape App Store in March 2014, has since registered more than 7,700 downloads, and has been highly rated by more than 25 users. In this paper, we present eXamine's new automation features that enable researchers to compose reproducible analysis workflows to generate visualizations of small, set-annotated graphs.

## Introduction

**Figure 1.**
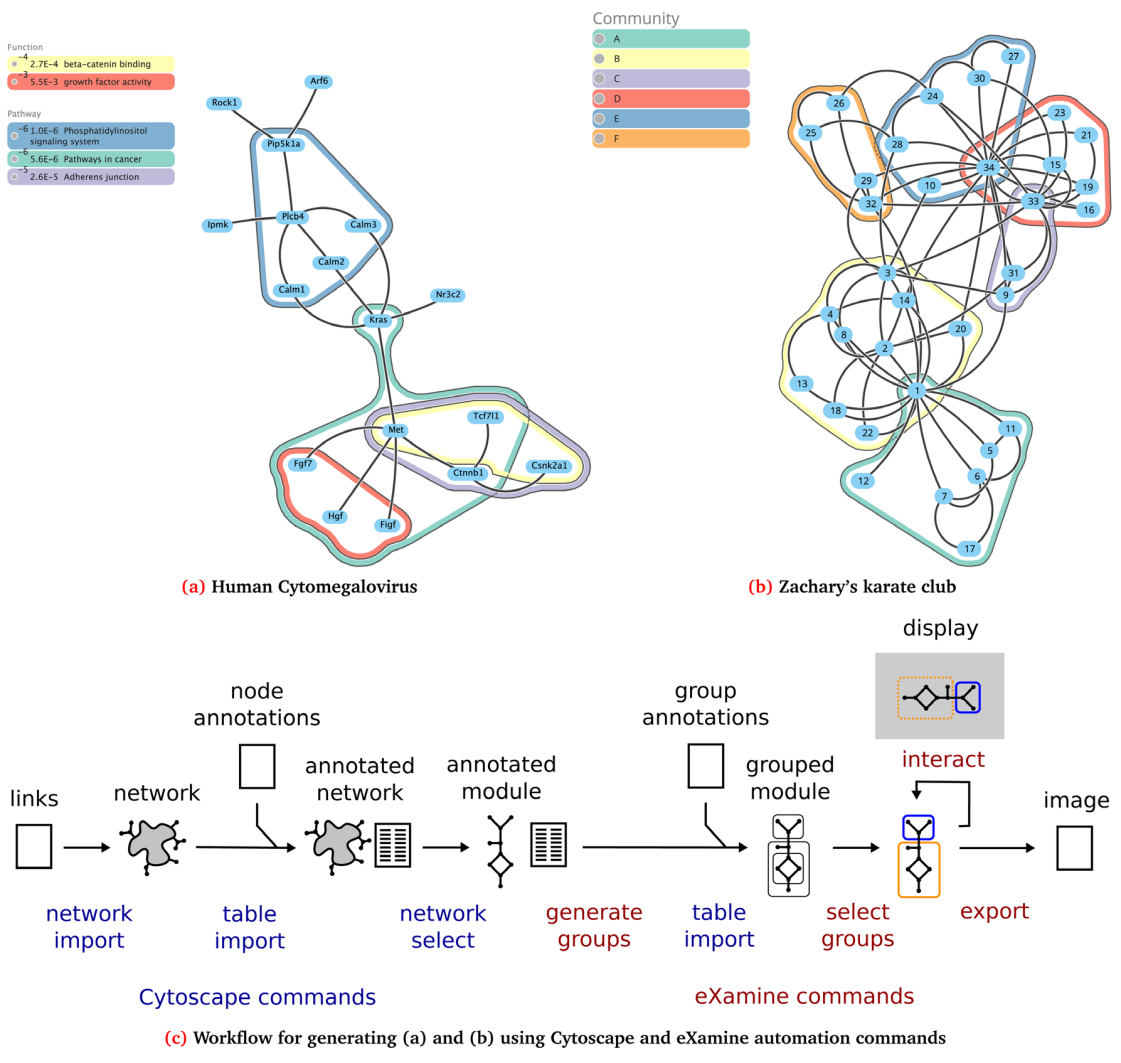
eXamine displays set membership as contours on top of a node-link layout. ( **a**– **b**) The graphs generated in the two use cases using eXamine’s automation features. ( **c**) The first step in the workflow consists of importing a network, followed by importing node annotations that associate each node with a set of groups. Next, we optionally select a smaller subnetwork to visualize. We generate an internal representation of the groups, and import additional group annotations. After selecting the groups to visualize, we export an image of the visualization. Alternatively, we can launch a window that allows the user to select different groups.

The Cytoscape app eXamine visualizes a small graph and a collection of node sets. The main purpose of eXamine is to aid in the interpretation of a small subnetwork module extracted from a large biological network ^1^. Cytoscape apps like jActiveModules ^2^ or external tools like Heinz ^3^ extract subnetwork modules from a protein-protein interaction network given gene expression data. To interpret the identified subnetwork module, a frequent follow-up analysis is to compute enrichment of the nodes of the identified module in terms of known annotations such as from the Gene Ontology (GO) ^4, 5^ or from the Kyoto Encyclopedia of Genes and Genomes (KEGG) ^6^. These annotations are a collection of node sets. eXamine provides a visual analysis approach that facilitates interpretation of the subnetwork module and the identified node sets by biologists. Given a small collection of node sets, eXamine generates a visualization of a small graph as a node-link layout together with contours for the selected sets ( Figure 1). More specifically, the layout is computed using a variation of the algorithm described in 7. This algorithm preserves topological distances along inter-module links as much as possible, while making sure that none of the nodes overlap. In addition, spanning graphs are derived for those sets that have been selected by the user. These graphs are included in the computation of topological distances between nodes, pulling the nodes closer together. The spanning graphs are also used to draw the set contours, by adjusting the associated links to form the rounded shapes that visually encompass and connect nodes.

As an alternative to eXamine, the existing group layout of Cytoscape can be used to show node partitions by visualizing disjoint sets in separate circles. The Venn and Euler diagram app ^8^ for Cytoscape visualizes overlapping sets. However, in both cases network and group analysis are visually separate. Finally, Cytoscape provides a group viewer ^I^ that aggregates groups into meta-nodes, without making group overlaps explicit. Here, we present a new version of eXamine that uses Cytoscape’s recently introduced automation features. With these new features it becomes possible to create reproducible analysis workflows that generate appealing visualizations of small, set-annotated graphs. We demonstrate eXamine’s new automation features using two use cases. The first use case replicates the case study provided in the original eXamine publication ^1^. The second use case, the analysis of a social network, demonstrates that eXamine is applicable to other domains beyond computational biology.

## Methods

### Implementation

eXamine is implemented in Java and is available as an app for Cytoscape 3. We used WebCola algorithms ^II^ to simultaneously lay out nodes, links, and set contours. We refer to the original publication ^1^ for additional implementation details regarding the used visualization techniques. Since the typical analysis workflows of eXamine consist of relatively simple commands that do not require streaming of complex data, we implemented the automation features through the ‘Command’ interface rather than the ‘Function’ interface. As a result, eXamine’s commands can be either directly from Cytoscape or through Cytoscape’s REST (CyREST) interface from a Jupyter notebook or from the programming language R.

### Operation

As eXamine requires Cytoscape 3.6 to run, eXamine has the same system requirements as Cytoscape 3.6. eXamine can be operated through the Cytoscape graphical user interface (GUI) or through the new ‘Command’ interface of Cytoscape. We refer to the original publication ^1^ for GUI instructions. In the following, we will describe and use the new ‘Command’ interface of eXamine.

Table 1 provides a summary of the API of the eXamine commands and their parameters. To enable workflow authors to use eXamine’s automation most effectively, we also generated Swagger-based documentation that describes the commands and arguments. This documentation can be accessed in the Cytoscape menu: Help → Automation → CyREST Command API. Figure 1 shows a typical workflow of eXamine analysis, where the commands provided are in the ‘examine’ namespace (red) and the commands provided by Cytoscape are colored blue.

**Table 1.** Application Programming Interface (API) of Cytoscape commands provided by eXamine. Figure 1 shows an example workflow using the below commands.

Command	Argument (type)	Description
**examine generate** **groups**		Generates eXamine groups from a given set of columns of the node table
	○ network (CyNetwork) ○ selectedGroupColumns (List) ○ useAllNodes (Boolean)	Network for which to generate the groups Columns of the node table from which the groups will be created (comma separated) Indicates whether to use all nodes for group generation, or only those nodes currently selected in Cytoscape
**examine select** **groups**		Select groups to be visualized by eXamine
	○ selectedGroup (List)	Group identifiers to select (comma separated)
**examine remove** **groups**		Removes all groups generated by eXamine
	○ network (CyNetwork)	Network from which to remove the groups
**examine update** **settings**		Updates visualization settings
	○ network (CyNetwork) ○ labelColumn (String) ○ urlColumn (String) ○ scoreColumn (String) ○ showScore (Boolean) ○ selectedGroupColumns (List)	Network to visualize Column of the node table that contains the node labels Column of the node table that contains URLs with additional information about the nodes Column that contains annotation enrichment scores Indicates whether to show annotation enrichment scores Group columns of the node table to show in the visualization
**examine interact**		Shows the selected network and groups in an interactive visualization window
**examine export**		Generates an SVG file with a visualization of the selected network and groups
	○ path (String)	Filename

### Use cases

To illustrate the new ‘Command’ interface of eXamine, we present two use cases. In the first use case, we describe a workflow to study a small subnetwork module extracted from the KEGG mouse network ^6^. In the second use case, we describe a workflow to study Zachary’s karate club, a well-known social network. Both workflows are available as Jupyter notebooks and as R markdown documents. The workflows require Cytoscape (≥ v3.6) and a recent Python version (Python v2.7 or Python v3.6) or R (≥ v3.4). We note that the commands described in the two use cases can also be directly executed from Cytoscape via the ‘Commands dialog’ or a separate ‘Commands script’ ^III^.

### Use case 1: Dysregulated signaling in Human Cytomegalovirus

The *Human Cytomegalovirus (HCMV)* is a highly-contagious herpes virus. Previously ^1^, we used eXamine to interpret a small subnetwork module (17 nodes and 18 edges) extracted from the KEGG mouse network using Heinz ^3^ given gene expression data of an HCMV-infected mouse cell line. Node sets of this subnetwork module were annotated using enriched pathways from KEGG and enriched terms from GO. Below, we provide Python code ^IV^ that uses Cytoscape’s and eXamine’s automation features to generate Figure 1. An R markdown document for this use case is available on the git repository ^V^.

1. To begin, we define a helper function that creates HTTP POST requests.

import requests
  PORT_NUMBER = 1234
  BASE = "http://localhost:" + str (PORT_NUMBER) + "/v1/"
  BASE_URL = "https://raw.githubusercontent.com/ls–cwi/"
           + "eXamine/master/doc/tutorial/"

  
def executeRestCommand(namespace="", command="", args={}):
      postString = BASE + "commands/" + namespace + "/" + command
      firstarg = True
      for arg in args:
          postString += ("?" if firstarg else "&") + arg + "=" + args[arg]
          if (firstarg): firstarg = False 
	res = requests.post(postString, json=args)
      return res
2. We import the full KEGG mouse network using the ‘network import url’ command provided by Cytoscape. This a protein-protein interaction network that is typically used for the analysis of high-throughput biological data in the context of a biological network. The Cytoscape app KEGGscape provides functionality for importing pathways from KEGG ^9^.

executeRestCommand("network","import url",
            {"indexColumnSourceInteraction" : "2",
            "indexColumnTargetInteraction" : "2",
            "url" : BASE_URL + "edges.txt"})
3. We import node annotation, containing set membership information, using the ‘table import url‘ command provided by Cytoscape. We note that the entries of the columns of type list (indicated by ‘sl’ in ‘dataTypeList’) are separated by a pipe character.

executeRestCommand("table","import url",
                {"firstRowAsColumnNames" : "true",
                "keyColumnIndex" : "1", "startLoadRow" : "1",
                "dataTypeList" : "s,s,f,f,f,s,s,s,sl,sl,sl,sl",
                "url" : BASE_URL + "nodes_induced.txt"})
4. Using the ‘network select’ command provided by Cytoscape, we select the nodes that comprise the subnetwork module identified by Heinz ^3^.

executeRestCommand("network","select",{"nodeList" : "Module:small"})
5. Using the ‘examine generate groups’ command, we generate group nodes for each member of the specified sets that occur in the module (the nodes we previously selected).

executeRestCommand("examine","generate groups",
                {"selectedGroupColumns" : "Function,Pathway"})
6. We import additional annotations that describe the generated groups using the ‘import table url’ command of Cytoscape.

executeRestCommand("table","import url",
                {"firstRowAsColumnNames" : "true",
                "keyColumnIndex" : "1", "startLoadRow" : "1",
                "url" : BASE_URL + "sets_induced.txt"})
7. As described in 1, we consider groups from the sets *Pathway* and *Function* using the ‘examine update settings’ command. In addition, we specify the columns that contain node labels and URLs as well as group scores and annotations.

executeRestCommand("examine","update settings",
                 {"labelColumn" : "Symbol", "urlColumn" : "URL",
                "scoreColumn" : "Score", "showScore" : "true",
                "selectedGroupColumns" : "Component, Function"})
8. Using the ‘examine select groups’ command, we select the same groups as described in 1.

executeRestCommand("examine","select groups",{"selectedGroups" :
                "GO:0008013,GO:0008083,mmu04070,mmu05200,mmu04520"})
9. Finally, we export the visualization to a scalable vector graphics (SVG) file using the ‘examine export’ command. Figure 1a shows the resulting graph.

executeRestCommand("examine","export", {"path": "fig1a.svg"})
  Alternatively, using the ‘examine interact’ command, we can launch an interactive visualization window that allows us to select different groups.

executeRestCommand("examine","interact", {})

### Use case 2: Zachary’s karate club

We consider the graph ‘Zachary’s karate club’, which is an undirected social network of friendships between 34 members of a karate club at a US university in the 1970s ^10^. Here, we use eXamine to visualize the six overlapping communities of this network identified in 11. We provide Python code below ^VI^. The corresponding R markdown document is available on the git repository ^VII^.

1. We use the same helper function as defined in the previous use case.
2. We import the network using the ‘network import url‘ command provided by Cytoscape.

executeRestCommand("network","import url",
                {"indexColumnSourceInteraction" : "2",
                "indexColumnTargetInteraction" : "2",
                "url" : BASE_URL + "edges_karate.gml"})
3. We import node annotation, containing set membership information, using the ‘table import url‘ command provided by Cytoscape. Entries of the column of type list (indicated by ‘sl’ in ‘dataTypeList’) are separated by a pipe character.

executeRestCommand("table","import url",
                {"firstRowAsColumnNames" : "true",
                "keyColumnIndex" : "1", "startLoadRow" : "1",
                "dataTypeList" : "s,sl",
                "url" : BASE_URL + "nodes_karate_induced.txt"})
4. Using the ‘network select’ command provided by Cytoscape, we select all nodes of the network.

executeRestCommand("network" ,"select", {"nodeList":"all"})
5. Using the ‘examine generate groups’ command, we generate group nodes for each member of the specified sets that annotate the nodes of the network.

executeRestCommand("examine","generate groups",
                {"selectedGroupColumns" : "Community"})
6. We consider groups from the set *Community* using the ‘examine update settings’ command.

executeRestCommand("examine","update settings",
                {"labelColumn" : "label", "urlColumn" : "label",
                "showScore" : "false",
                "selectedGroupColumns" : "Community"})
7. We select all six groups using the ‘examine select groups’ command.

executeRestCommand("examine","select groups",
                {"selectedGroups" : "A,B,C,D,E,F"})
8. Finally, we export the visualization to an SVG file using the ‘examine export’ command. Figure 1b shows the resulting graph.

executeRestCommand("examine","export", {"path": "fig1b.svg"})

## Discussion and conclusions

eXamine is limited to visualizing small, relatively sparse networks. It is not possible to use eXamine to construct a comprehensive layout if the network consists of hundreds of nodes or if there are dozens of annotation sets to visualize at the same time. This is a natural limitation of any visualization approach based on node-link diagrams and set contours.

eXamine currently uses Cytoscape’s CyREST API to import networks and their annotations. If apps that provide gene enrichment analysis functionality, such as BiNGO ^12^, would expose this functionality through the CyREST API, we envision updating the workflow to include this type of upstream analysis. Finally, it would be good to enhance the API to return richer R and Python data types rather than a flag indicating whether the command succeeded. For instance, upon a ‘generate groups’ it would be good to actually return a dataframe containing the groups. This, however, will require switching from the ‘Command’ interface to the ‘Functions’ interface.

## Summary

eXamine is a Cytoscape app for a set-oriented visual analysis approach for small annotated graphs that displays set membership as contours on top of a node-link layout ( Figure 1). In this paper, we presented new automation features for eXamine that are accessible through Cytoscape’s REST API ( Table 1). As such, researchers can embed eXamine in reproducible and well-documented workflows that generate appealing visualizations of small, set-annotated graphs ( Figure 1). We demonstrated two such workflows in the context of computational network biology and social network analysis.

## Data and software availability

1. Cytoscape app store: http://apps.cytoscape.org/apps/examine

2. Source code: https://github.com/ls-cwi/eXamine

3. Link to latest commit as of release: https://github.com/ls-cwi/eXamine/commit/c1c3e643250c668c77a40cb982f9d122993e22a5

4. Link to archived source code as at time of publication: https://doi.org/10.5281/zenodo.1302788 ^13^

5. License: GPL v2.0

6. Link to use case data: https://github.com/ls-cwi/eXamine/blob/master/doc/tutorial.

7. Link to Jupyter notebooks: https://github.com/ls-cwi/eXamine/blob/master/doc/tutorial/eXamineNotebook/eXamineTutorial.ipynb and https://github.com/ls-cwi/eXamine/blob/master/doc/tutorial/eXamineNotebook/eXamineTutorial2.ipynb.

8. Link to R markdown documents: https://github.com/ls-cwi/eXamine/blob/master/doc/tutorial/eXamineNotebook/eXamineTutorial_R.Rmd and https://github.com/ls-cwi/eXamine/blob/master/doc/tutorial/eXamineNotebook/eXamineTutorial2_R.Rmd.

## Notes

^I^ http://manual.cytoscape.org/en/stable/Creating_Networks.html#grouping-nodes

^II^ http://ialab.it.monash.edu/webcola/

^III^ http://manual.cytoscape.org/en/stable/Programmatic_Access_to_Cytoscape_Features_Scripting.html

^IV^ https://github.com/ls-cwi/eXamine/blob/master/doc/tutorial/eXamineNotebook/eXamineTutorial.ipynb

^V^ https://github.com/ls-cwi/eXamine/blob/master/doc/tutorial/eXamineNotebook/eXamineTutorial_R.Rmd

^VI^ https://github.com/ls-cwi/eXamine/blob/master/doc/tutorial/eXamineNotebook/eXamineTutorial2.ipynb

^VII^ https://github.com/ls-cwi/eXamine/blob/master/doc/tutorial/eXamineNotebook/eXamineTutorial2_R.Rmd
