# Supplementary material for: eXamine: Visualizing annotated networks in Cytoscape
Source: F1000Res. 2018 Jul 5;7:519. Originally published 2018 Apr 30. [Version 2] doi: 10.12688/f1000research.14612.2 (PMC6013758; doi:10.12688/f1000research.14612.2)
Supplement: Peer review history

# Peer review: Referee response for version 2

**DOI:** 10.5256/f1000research.16899.r35797

**Published:** 2018-07-19

**Authors:** Richa Batra

**Affiliations:** 1 Institute of Computational Biology, Helmholtz Center Munich, Neuherberg, Germany; 2 Technical University of Munich, Munich, Germany

Thank you for updating the manuscript and adding the R code.

I have read this submission. I believe that I have an appropriate level of expertise to confirm that it is of an acceptable scientific standard.

# Peer review: Referee response for version 2

**DOI:** 10.5256/f1000research.16899.r35798

**Published:** 2018-07-13

**Authors:** Alexander Pico

**Affiliations:** 1 Gladstone Institutes, San Francisco, CA, USA

Thanks for addressing the points of my review.

I have read this submission. I believe that I have an appropriate level of expertise to confirm that it is of an acceptable scientific standard.

# Peer review: Referee response for version 2

**DOI:** 10.5256/f1000research.16899.r35796

**Published:** 2018-07-10

**Authors:** Markus List

**Affiliations:** 1 Chair of Experimental Bioinformatics, TUM School of Life Sciences, Technical University Munich, Munich, Germany

I have no additional comments to make.

I have read this submission. I believe that I have an appropriate level of expertise to confirm that it is of an acceptable scientific standard.

# Peer review: Referee response for version 2

**DOI:** 10.5256/f1000research.16899.r35799

**Published:** 2018-07-06

**Authors:** Barry Demchak

**Affiliations:** 1 Department of Medicine, University of California, San Diego, La Jolla, CA, USA

Thank you. These changes satisfy my concerns. Thank you for taking such care with this material.

I have read this submission. I believe that I have an appropriate level of expertise to confirm that it is of an acceptable scientific standard.

# Peer review: Referee response for version 1

**DOI:** 10.5256/f1000research.15901.r33592

**Published:** 2018-06-25

**Authors:** Alexander Pico

**Affiliations:** 1 Gladstone Institutes, San Francisco, CA, USA

Many researchers arrive at point in enrichment analysis where they have tables of enriched ontology terms and pathways, but don't know how to go beyond presenting those data as tables or perhaps bar graphs. The use of networks is an intuitive and powerful visualization option. The eXamine app for Cytoscape provides a nice approach for displaying a simplified version of enrichment results. A couple steps are missing, however, and a few minor edits are suggested.

 Missing steps:

1. All sources of enrichment that I'm aware of provide rows of terms and a list of genes in a single field per row.  It would be nice if you provided a bit of Python to transform that common input data type to that required by eXamine.
2. I was wondering about genes that belong to more than one term (often the case). I looks like your tool handles these via a piped list. Is that right? Please describe the correct syntax in the text so readers don't have to guess.

Minor suggestions:

1. Groups in Cytoscape are no longer provided as a separate plugin, but rather are now integrated into the core of Cytoscape. Your reference to "RBVI Cytoscape plugins" should be updated to simply mention the Cytoscape manual and point to the section on groups: http://manual.cytoscape.org/en/stable/Creating_Networks.html#grouping-nodes
2. The LaTeX formatting for your Python code is unfortunately rendering single quotes in a way that throws SyntaxError in Python. Can you update the rendering so one can simply copy/paste snippets?
3. In the next version of this paper, I'd highly recommend using py2cytoscape ( https://github.com/cytoscape/py2cytoscape). This will make the code you have write much more concise and easier to maintain or adapt. Likewise, I'd really like to see R examples and you can leverage RCy3 to make that code easy to write as well ( http://bioconductor.org/packages/release/bioc/html/RCy3.html).

I have read this submission. I believe that I have an appropriate level of expertise to confirm that it is of an acceptable scientific standard, however I have significant reservations, as outlined above.

# Response

**Published:** 2018-07-01

**Authors:** Mohammed El-Kebir

**Affiliations:** University of Illinois at Urbana-Champaign, USA

We thank the referee for the positive feedback. Below our point-by-point response to the referee's comments.

- Regarding the missing details on generating the enrichment input, the DAVID webtool ( https://david.ncifcrf.gov/) yields a file where the rows are genes containing a list of terms. Implementing a generic Python function that transforms enrichment output that comes in various to our required format is a challenging task. Ideally, the enrichment step should be part of the workflow, as described in Discussion.
- We handle genes with multiple terms using lists, and the default separator used by Cytoscape’s import table functionality is indeed a pipe character. We updated the tutorials and text to describe this.
- We updated the reference to "RBVI Cytoscape plugins".
- We replaced all single quotes by double quotes, which are retained by the used LaTeX package for displaying the code (package is ‘listing’). Simple copy-paste of the commands into the Python interpreter should work now.
- We thank the referee for the suggestions regarding R and Python. We now use the RCy3 library for the two use cases in R. As for py2cytoscape, we found that the functionality that we need is not supported directly and requires separate script files for each command. We found that using the REST interface directly was easier.

# Peer review: Referee response for version 1

**DOI:** 10.5256/f1000research.15901.r34808

**Published:** 2018-06-21

**Authors:** Markus List

**Affiliations:** 1 Chair of Experimental Bioinformatics, TUM School of Life Sciences, Technical University Munich, Munich, Germany

In their manuscript “eXamine: Visualizing Annotated Networks in Cytoscape”, Spohr et al. describe a new version of their Cytoscape tool eXamine. eXamine allows user to enrich the visualization of small networks via colored contours which represent specific annotations of subnetworks, e.g. functional annotation. eXamine is very useful because it not only highlights group properties in a visually appealing way but also allows the representation to be changed interactively. The software always attempts to achieve an optimal layout in which overlaps are kept at a minimum. Here, the authors have adapted eXamine to make use of the new Cytoscape automation features which allow for programmatic control of the plotting process, thus allows for generating reproducible results that can further be embedded in scripts.

 The manuscript is well written and describes the new features and their usage clearly.

 The code of eXamine is hosted on github and contains the two use cases from the manuscript in the form of popular Jupyter notebooks. Each step of the examples is well described and illustrates to the user how eXamine can be used in a scripting environment such as Python. In particular less obvious steps like extracting network ids from a JSON result help new users to achieve results fairly quickly.

 I have only minor comments:

- Unfortunately the second use case (Jupyter notebook) in the git repository did not work due to the use of a local file path instead of a public URL.
- Add the end of the first paragraph the manuscript says “by dilating and eroding the associated links”. I’m not sure what is meant here with erosion.
- Use case 1, first paragraph, there is a typo: feautures -> features
- The github readme should briefly describe the jupyter notebooks and link to them.

I have read this submission. I believe that I have an appropriate level of expertise to confirm that it is of an acceptable scientific standard.

# Response

**Published:** 2018-07-01

**Authors:** Mohammed El-Kebir

**Affiliations:** University of Illinois at Urbana-Champaign, USA

We thank the referee for the positive feedback. Below our point-by-point response to the referee's comments.

- We fixed the link to the git repository for the second use case.
- We changed the wording of the sentence where we previously used 'erosion'.
- We fixed the typo. Thank you for pointing this out.
- We updated the Github README.md file.

# Peer review: Referee response for version 1

**DOI:** 10.5256/f1000research.15901.r34810

**Published:** 2018-06-20

**Authors:** Richa Batra

**Affiliations:** 1 Institute of Computational Biology, Helmholtz Center Munich, Neuherberg, Germany; 2 Technical University of Munich, Munich, Germany

This paper presents new features to an existing cytoscape app namely eXamine. eXamine was initially developed to visualize small graphs and node sets. This new version of the app has automation features for reproducible research. Given the importance of reproducible research, this new version of the app is highly relevant. 

 Figure 2 should really be Figure 1. For someone new to eXamine, it aptly summarizes the app. Also the summary at the end is very clear. 

 It would have been great to have a small dataset and step by step guide through the cytoscape app. Then small R script that I can run to generate the same results. Could you extend it for R users? Do you plan it in future work?

 If the paper is about automation of eXamine via python, then it should reflect in title. The current name of the paper is misleading.

I have read this submission. I believe that I have an appropriate level of expertise to confirm that it is of an acceptable scientific standard, however I have significant reservations, as outlined above.

# Response

**Published:** 2018-07-01

**Authors:** Mohammed El-Kebir

**Affiliations:** University of Illinois at Urbana-Champaign, USA

We thank the referee for the positive feedback. Below our point-by-point response to the referee's comments.

- We have merged Figures 1 and 2 in the revised manuscript.
- We included R example code in the revised manuscript.

# Peer review: Referee response for version 1

**DOI:** 10.5256/f1000research.15901.r33593

**Published:** 2018-05-18

**Authors:** Barry Demchak

**Affiliations:** 1 Department of Medicine, University of California, San Diego, La Jolla, CA, USA

This article describes the use of new Commands exposed by the eXamine app. 

 The article is well written and adequately describes the Commands. My comments are advisory, and I hope they can improve the paper.

 The procedure for using Commands should be well known to a proficient Cytoscape user. However, there's an important class of user that wants to use Automation but isn't a Cytoscape expert. To help him/her along good reference Cytoscape manual (Commands Tool), and good to point out that the commands can be used from the Commands dialog, a separate Commands script (via the Cytoscape command line or the Tools | Execute Command File menu item), or via Python. (See last paragraph of Methods section.) Good, too, to remind the user that eXamine commands are in the examine namespace, and that different namespaces resolve to Cytoscape and other apps.

 This paper is about Python usage. Good to remind user that similar calls can be made from R.

 In the Introduction, it would be helpful to justify eXamine automation by giving an example of workflows that become possible. This motivates the user. This could be as simple as summarizing Use cases.

 For Discussion, it may be worth speculating on the value of providing Python and R libraries that act as cover functions for the eXamine REST calls. Python and R programmers really want to think in terms of Python and R, not REST.

 The color coding in Figure 2 is very effective. Can you also color code the corresponding REST calls in the Use Case section? This may require changing the color scheme so the examples can be easily read. (Maybe Red/Green or Red/Blue??)

 In Use Cases, good to say where the Jupyter notebooks are ... even if you identify them at the end of the paper.

 As a side note, I notice that all eXamine endpoints are GET. The Commands convention we're using now is POST, with GET being deprecated. In this case, it doesn't matter much, as the parameter list isn't long and there isn't any return result that would benefit from JSON encoding. For a future release, good to consider POST versions, too. That way, if an error occurs, you'll be able to return it in a CIResponse structure.

 For the BASE_URL, the datasets are necessary to run the examples. Can you list them at the end of the paper? Of course, they must be persistently available for the life of the paper, correct??

 In Use Case 1, step 2, it would be good to give a sentence or two explaining how/why a user would have created this dataset.

 That's it ... nice job!

I have read this submission. I believe that I have an appropriate level of expertise to confirm that it is of an acceptable scientific standard, however I have significant reservations, as outlined above.

# Response

**Published:** 2018-07-01

**Authors:** Mohammed El-Kebir

**Affiliations:** University of Illinois at Urbana-Champaign, USA

We thank the referee for the positive feedback. Below our point-by-point response to the referee's comments.

- We included the following sentences along with a reference to the Cytoscape documentation:
*‘Figure 1 shows a typical workflow of eXamine analysis, where the commands provided are in the `examine' namespace (red) and the commands provided by Cytoscape are colored blue.’*
**
* ‘We note that the commands described in the two use cases can also be directly executed from Cytoscape via the `Commands dialog' or a separate `Commands script'. ^3^’*
- We now provide R code for the two use cases.
- We reworded the last paragraph of the introduction to more clearly describe the benefits of eXamine's new automation features.
- We wrote the following sentences in Discussion:
*‘Finally, it would be good to enhance the API to return richer R and Python data types rather than a flag indicating whether the command succeeded. For instance, upon a `table import' it would be good to actually return a dataframe. This, however, will require switching from the `Command' interface to the `Function' interface.’*
- Thank you for the excellent suggestion about the coloring. We colored eXamine commands red and Cytoscape commands blue.
- We included URLs to the Jupyter and R markdown documents.
- We now use POST commands.
- We now include links to the datasets. They are indeed persistently archived using zenodo.
- We included the following sentences in step 2 of use case 1:
*‘This a protein-protein interaction network that is typically used for the analysis of high-throughput biological data in the context of a biological network. The Cytoscape app KEGGscape provides functionality for importing pathways from KEGG [9].’*
